# A New Target in Inflammatory Diseases: Lycopene

**DOI:** 10.5152/eurasianjmed.2022.22303

**Published:** 2022-12-01

**Authors:** Zeynep Karaköy, Elif Cadirci, Busra Dincer

**Affiliations:** 1 Department of Pharmacology, Erzincan Binali Yildirim University, Faculty of Pharmacy, Erzincan, Turkey; 2 Department of Pharmacology, Ataturk University, Faculty of Medicine, Erzurum, Turkey

**Keywords:** Lycopene, free radicals, inflammation

## Abstract

Inflammation is a response to various injuries, illnesses, and severe trauma. The primary function of inflammation is to combat pathogens, eliminate them from the body, and initiate wound healing. However, inflammation also contributes to numerous diseases, such as cancer, cardiovascular disease, diabetes, obesity, osteoporosis, rheumatoid arthritis, inflammatory bowel disease, and asthma. As the importance of nutrition in maintaining human health has become increasingly recognized, the consumption of natural antioxidants has gained popularity, especially in developed countries. A growing body of research has shown that consuming foods rich in lycopene can protect individuals from a range of conditions, including cancer, heart disease, and other diseases. As a result, lycopene is gaining recognition as a potential protective antioxidant in the fields of medicine and pharmacology. This review aims to highlight the effects of lycopene on inflammatory diseases and provide a foundational understanding for researchers interested in further research on lycopene.

## Introduction

Inflammation is the body’s primary immune response to harmful stimuli such as allergens and pathogens.^1^ In addition to killing pathogens, it initiates tissue repair processes and helps restore homeostasis in damaged areas.^2,3^ These stimuli trigger the body’s inflammatory processes that begin with the expression of pro-inflammatory cytokines and chemokines. Cytokines activate endothelial cells,^4^ leading to increased vascular permeability and facilitating the entry of immune cells into tissues at the site of inflammation.^5^

Furthermore, chemokines attract eosinophils, neutrophils, monocytes, and mast cells to the injury site. Once the infection has been cleared, mechanisms are activated to prevent further damage to the host and begin tissue repair. These control mechanisms increase the expression of anti-inflammatory cytokines to end inflammation and inhibit the expression of pro-inflammatory cytokines. Eventually, the inflammation subsides as inflammatory cells undergo apoptosis, and the tissue returns to homeostasis.^6^

## Inflammatory Mediators

### Pro-Inflammatory Cytokines

Cytokines are substances in peptide or glycoprotein structure with a weight of 20-30 kD, mainly synthesized from immune cells, including monocytes, macrophages, and lymphocytes.^7^ Cytokines are grouped as pro-inflammatory^7^ or anti-inflammatory^8^: interleukin (IL)-1, IL-6, IL-12, IL-1β, interferon (IFN)-γ, tumor necrosis factor (TNF)-α, and granulocyte-macrophage colony-stimulating factor are proinflammatory cytokines.^5^ At the same time, IFN-α, IL-4, IL-10, IL-13, and transforming growth factor-β are anti-inflammatory cytokines.^6,9^

Tumor necrosis factor-α is an important pro-inflammatory cytokine of the TNF superfamily, abundantly produced by immune cells during acute inflammation.^3^ Tumor necrosis factor-α causes vasodilation and increased vascular permeability, leading to systemic edema.^10^

### Nuclear Factor Kappa-B

**Figure 1.**
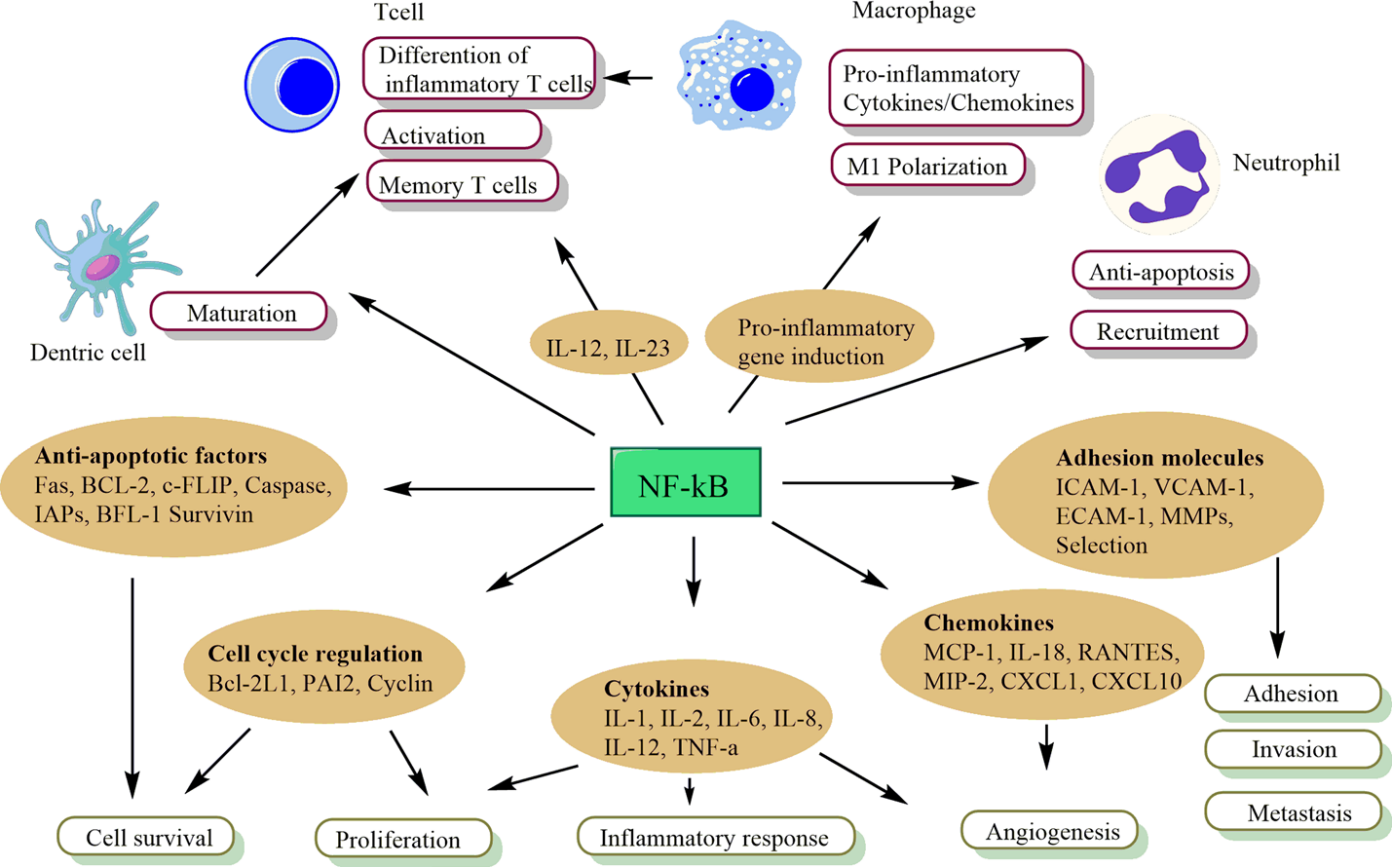
Nuclear factor kappa-B and inflammatory response (Adapted from Ref. 7).

Nuclear factor kappa-B (NF-κB) is an inducible transcription factor that regulates the expression of cytokines involved in inflammation.^11^ When activated, NF-κB stimulates the transcription of various inflammatory genes. It plays a significant role in the inflammatory response by directly increasing the production of inflammatory cytokines, chemokines, and adhesion molecules; influencing T cell activation, differentiation, and function; and regulating cell proliferation, apoptosis, and morphogenesis^12^ (see Figure 1).

### C-Reactive Protein

C-reactive protein (CRP) is an acute-phase protein that is synthesized by the liver. C-reactive protein is found at very low levels in healthy individuals. C-reactive protein levels increase during infections and inflammatory diseases.^13^ Its primary function is to coat damaged cells in inflammation, enabling them to be recognized by other immune cells. As such, CRP is a systemic marker of inflammation.^3^

### Reactive Oxygen Species

Stimulating these inflammatory cells by tumor promoters, parasites, bacteria, or particles releases reactive oxygen species (ROS). After reaching the site of inflammation, leukocytes are involved in the process of phagocytosis, which is accompanied by a process called “respiratory burst.” That is, they engulf bacteria or foreign particles. At this stage, leukocytes rapidly increase their oxygen uptake. As a result, ROS are produced, such as superoxide anion, lipid peroxyl, hydrogen peroxide, alkoxyl, and hydroxyl radicals. Research has shown that the release of ROS increases the expression of intercellular cell adhesion molecules (ICAM), activates NF-κB, and increases inflammation.^14^

### Lycopene

Lycopene, a red carotenoid, was first discovered by Millardet in 1876 as a red pigment in tomatoes, later named “lycopene” by Schunck.^15^ It is predominantly found in carrots, watermelon, papaya, gac fruit (red), asparagus, and parsley.^16^ Among the various common carotenoids, lycopene has the strongest antioxidant properties.^17^ Lycopene is a natural antioxidant and anti-inflammatory agent that can potentially prevent and treat diseases.^18,19^

### Physicochemical Properties of Lycopene

**Figure 2.**
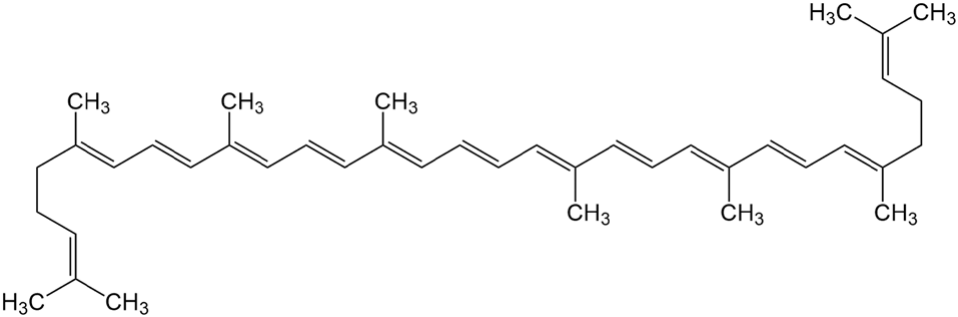
Molecular structure of lycopene.

It is a symmetrical tetraterpene composed of 40 carbon and 56 hydrogens (C_40_H_56_) and an isoprene unit (Figure 2). Lycopene is an acyclic isomer of β-carotene,^20^ but unlike β-carotene, lycopene lacks the β-ionic ring structure. Therefore, there is no provitamin A activity. On the other hand, lycopene is a much higher antioxidant than β-carotene and α-tocopherol (vitamin E).^21^

## Therapeutic Potential of Lycopene

### Antioxidant Effect

Lycopene is a powerful and important antioxidant. It prevents the oxidation of proteins, lipids, and deoxyribonucleic acid through its action on free radicals.^15,22,23^ Free radicals are in excess at the site of inflammation, and these free radicals cause tissue damage. Several studies have found that lycopene can modulate ROS levels.^24^ The antioxidant activity of lycopene comes from the conjugated double bond in its structure.^25^ Lycopene interacts chemically with ROS and prevents ROS-induced cell damage.^6^

### Anticancer Effect

Lycopene inhibits the expression of cyclin D1 in G0/G1 phases and arrests the cell cycle.^26^ Studies have shown that the level of lycopene in serum and tissues can inhibit the growth of cancer cells in different organs and reduce their carcinogenesis.^16^ The anti-inflammatory activity of lycopene is considered a key factor in suppressing the progression of carcinogenesis.^27^

### Possible Side Effect

Lycopenemia is a pigmentation disorder caused by the accumulation of lycopene. The skin color changes to a yellow-orange color. It is caused by the accumulation of lycopene due to excessive consumption of lycopene-rich foods. This accumulation occurs in the stratum corneum with a high lipid content and affinity for lycopene. The symptoms of lycopenemia typically disappear when the dietary intake of lycopene is reduced.^28^

### Lycopene and Inflammation

Lycopene is one of the most studied natural compounds due to its antioxidant, anti-inflammatory, and anticancer properties.^29,30^ It positively affects inflammation as it activates the expression of antioxidant genes and regulates the signaling pathways responsible for inducing inflammatory mediators.^15^

### Anti-Inflammatory Effect Mechanism of Lycopene

Lycopene generally exerts its anti-inflammatory activity through the following mechanisms:

1. Down-regulation of pro-inflammatory cytokines such as IL-1, IL-6, and TNF-α.
2. Reduction of other pro-inflammatory mediators such as inducible nitric oxide synthase (iNOS) and cyclooxygenase 2 (COX-2) expression.
3. Reducing the expression of NF-κB.^29^

**Figure 3.**
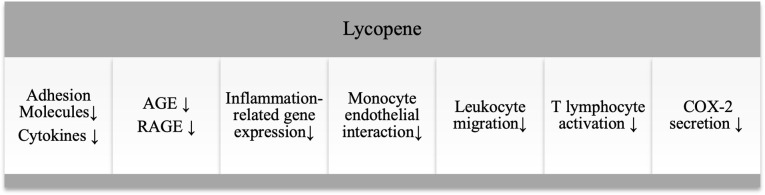
Anti-inflammatory effects of lycopene (Adapted from Ref. 25). AGE, advanced glycation end products; COX-2, cyclooxygenase 2; RAGE, AGE receptors.

Inhibiting the extracellular signal-regulated kinase (ERK) and p38 mitogen-activated protein (MAP) kinase in macrophages is another way to reduce inflammation.^29^ Figure 3 illustrates the anti-inflammatory effects of lycopene.

**Figure 4.**
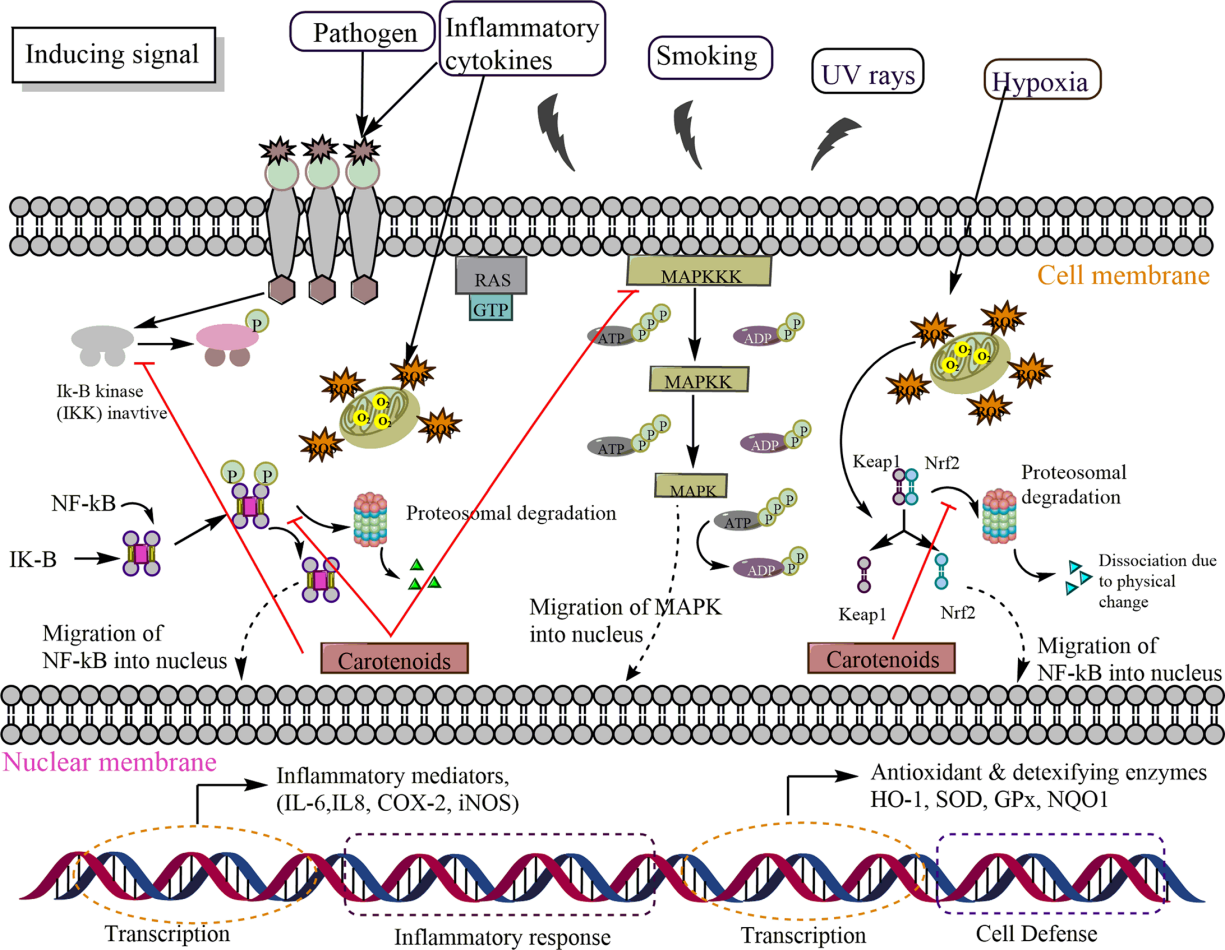
Anti-inflammatory effect of carotenoids on different inflammatory signaling pathways (Adapted from Ref. 22).

Lycopene has an important role in suppressing the inflammatory response. Its effect in this direction is mainly related to the inhibition of ROS production. In addition, lycopene inhibits the synthesis and expression of pro-inflammatory cytokines, including IL-1, IL-1β, IL-6, and TNF-α.^31^ Figure 4 shows the schematic of the anti-inflammatory effect of carotenoids on different inflammatory signaling pathways.

### In-Vivo/In Vitro Lycopene Research

Long-standing and unresolved inflammation is closely associated with many diseases, including rheumatoid arthritis (RA),^32^ inflammatory bowel disease (IBD), atherosclerosis,^33^ chronic obstructive pulmonary disease (COPD),^34^ asthma, neurodegenerative diseases, and cancer.^35^ Many natural compounds, including lycopene, are used to treat inflammation and inflammatory diseases.^9,36-40^ In vivo and in vitro studies with lycopene are summarized in Table 1.

## Lycopene in Inflammatory Diseases

### Anti-Inflammatory Effect of Lycopene on Asthma and Chronic Obstructive Pulmonary Diseases

Asthma is a heterogeneous disease caused by inflammation of the airways. It is characterized by airway hypersensitivity to stimuli, airway edema, and increased mucus secretion.^41^

Chronic obstructive pulmonary disease is a progressive, highly mortal, and morbid disease characterized by airway restriction, associated with an abnormal inflammatory response to exposure to risk factors. Inflammation in COPD affects the entire system, not just the airways.^42^ The disproportion in the oxidant–antioxidant system is one of the events leading to the onset of inflammatory reactions in COPD. Oxidative stress and the formation of free radicals infect airway epithelial cells, causing inflammation and obstruction in the airways.^42^

Lee et al^43^ showed that eosinophils in the lung stimulate T helper type 2 (Th2) cell responses. Therefore, Th2 cells are abundant in the airways, and Th2 cytokines play a very essential role in the pathophysiology of asthma. In addition, matrix metalloproteinase-9 induces the migration of lymphocytes, eosinophils, and neutrophils across basement membranes during tissue injury and repair.

Campos et al^44^ showed in both in vitro and in vivo experiments that lycopene inhibits TNF-mediated activation of the NF-κB signaling pathway and reduces the expression of inflammatory cytokines and chemokines.

### Anti-Inflammatory Effect of Lycopene on Rheumatic Diseases

Rheumatoid arthritis is a common, systemic, and chronic inflammatory disease that causes arthritis in the joints symmetrically. The disease affects about 1% of the world’s population, and if left untreated, can lead to joint damage, deformity, and related disability.^45^ Disruption of antioxidant systems in RA and oxidative stress caused by free radicals have an essential role in the etiology of RA.^46^ Moia et al^47^ reported that nanolycopene might be used as an anti-inflammatory agent to treat RA.

### Anti-Inflammatory Effect of Lycopene on Cardiovascular Diseases

Inflammation is associated with atherosclerosis, arterial stiffness, and major cardiovascular disease.^48^ Oxidative stress is recognized as a cardiovascular risk factor.^49,50^ Some research has shown that natural compounds with antioxidant and anti-inflammatory effects may be beneficial in preventing and treating cardiovascular diseases.^51^

Studies have also shown that lycopene can inhibit TNF-α-induced NF-κB activation, expression of ICAM-1, and the interaction between endothelial cells and monocytes. In addition, lycopene reduces the secretion of metalloproteinases by macrophages and inhibits the activation of T lymphocytes. Recently, lycopene has been an effective antiglycation agent, which can reduce the synthesis of advanced glycation end products (AGE) and AGE receptors (RAGE) and further contribute to vascular protection.^6,52^

An in vivo study reported the benefits of lycopene in preventing transplant vasculopathy by showing that intimal hyperplasia and smooth muscle cell proliferation and infiltration of inflammatory cells in allograft vessels were reduced by lycopene administration.^53^

Oxysterols, which result from the auto-oxidation of cholesterol, accumulate in the subendothelial artery layer and support the atherosclerotic process by playing an oxidative and pro-inflammatory role. Lycopene reduces atherosclerotic plaque formation by disrupting oxysterol-induced pro-inflammatory cytokine production and ROS production in macrophages.^52,54^

Considering the aforementioned anti-inflammatory mechanisms (reduction of AGE and RAGE synthesis, inhibition of proinflammatory cytokines and genes involved in inflammation, reduction of adhesion molecules, and downregulation of COX-2), lycopene may be helpful in the treatment of vascular inflammatory disorders.^52^

### Anti-Inflammatory Effect of Lycopene on Pancreatitis

Pancreatitis is a gastrointestinal disease with significant morbidity and mortality despite modern diagnosis and treatment methods. The severity of the disease mainly depends on the inflammatory responses that occur in the early phase of the disease. Many studies have reported that many natural compounds with antioxidant and anti-inflammatory properties protect pancreatitis.^55,56^

In an experimental pancreatitis model, cerulein binds to cholecystokinin (CCK) receptors and temporarily elevates intracellular Ca^2+^ levels. Increasing Ca^2+^ activates nicotinamide adenine dinucleotide phosphate (NADPH) oxidase, which produces ROS. Reactive oxygen species with increased levels induce the NF-κB signaling pathway and the expression of inflammatory cytokines (IL-1β, IL 6, and TNF-α). Thanks to its antioxidant properties, lycopene suppresses NF-κB activation and inhibits the inflammatory cytokine IL-6 in pancreatic acinar cells.^57,58^

In a study on rats, lycopene was found to decrease the levels of the inflammatory cytokines TNF-α and IL-1β, as well as the enzyme myeloperoxidase, and increase the levels of the antioxidant glutathione.^59^

### Anti-Inflammatory Effect of Lycopene on Inflammatory Bowel Diseases

Inflammatory bowel disease is a recurrent and chronic immune system disorder of unknown etiology that affects millions of people.^60^ Inflammatory bowel diseases are of 2 types: ulcerative colitis and Crohn's disease. Activation of NF-κB induces the expression of many molecules (such as IL-1β, TNF-α, IL-6, IL-8, ICAM-1) involved in the pathogenesis of IBD.^61^

Corticosteroids and 5-aminosalicylates are used in classical treatment. Immunosuppressive and biological agents are used in patients who do not respond to conventional treatment.^36^

However, corticosteroids are not considered an appropriate option in the long term, especially in children, due to their potentially serious side effects, such as osteopenia, growth retardation, and insulin resistance.^62^

Antioxidant and anti-inflammatory natural compounds have been used to treat IBDs.^63^ Studies have reported that lycopene alleviates inflammation in colitis. Lycopene treatment aims to reduce symptoms and remission, improve quality of life, and prevent complications.^64^

## Conclusion

Lycopene is an important antioxidant compound in eliminating free radicals released from oxidative stress due to various factors. It not only defends cells from free radical damage but also supports the bonds between cells and develops cell metabolism.

Given its numerous preventive properties that reduce the risk of oxidative stress-related diseases such as atherosclerosis, cardiovascular disease, neurodegenerative disorders, and cancer, it is important for human health and a balanced diet to include foods rich in lycopene.

This review study examined the relationship between lycopene, a bioactive component with different pharmacological benefits such as anticancer,^27^ antioxidant,^44^ cardioprotective,^31^ and antihypertensive effects, and the onset and progression of inflammation, as well as the potential role of lycopene in managing these diseases.

There are still many unknowns about the biological function of lycopene, and further research is needed to determine whether the metabolites of lycopene have any biological effects.

## Figures and Tables

**Table 1.** In Vitro/In Vivo Studies About the Lycopene’s Anti-Inflammatory Properties

In Vitro/In Vivo	Dose	Result	Reference
SW480 human colorectal cancer cells	10, 20, and 30 μM Lycopene	↓ COX-2, mRNA expression, and PGE2 production	65
Prostate cancer cells, LNCaP, PC3, and DU145	Lycopene	↓ IL-1, IL-6, IL-8, TNF-α expression	66
Rat pancreatic acinar cell line	5 µmol/L Lycopene	ROS, IL-6, NF-κB signaling activation ↓	58
J774A.1 macrophage cell line	0.5, 1.0, and 2.0 μM Lycopene	↓ TNF-α, IFN-γ	44
Airway epithelial cells (Calu-3 cells)	0, 2.5, 5, 10, and 25 μg/mL Lycopene	↓ NF-κB inactivation↓ IL6	67
RAW 264.7 cells	1–10 μM Lycopene	↓ IL-6	17
HUVEC	20 μM Lycopene	↓ CD14, TLR4, TNF-α, and NF-κB	68
Peripheral blood mononuclear cells (PBMC)	0.25, 0.5, 1.0, 2.0, and 4.0 μM Lycopene	↓ TNF-α and IL-1β; ↑ IL-10, IL-2, and IFN-γ;	69
Male BALB/c mice	4 mg Lycopene in 200 μL of water	↓ Eosinophils; ↓ IL-4, IL-5, IL-13	70
Male BALB/c mice	10 mg/kg/day Intragastric for 4 weeks	↓ TNF-α, NF-κB, and IL-1β	71
l-arginine-induced acute pancreatitis in rats	50 mg/kg Lycopene	↓ TNF-α	72
Severe acute pancreatitis from sodium taurocholate in rats	10 mg/kg Lycopene	↓ TNF-α, IL-6 ↓ NF-κB p65	73

COX-2, cyclooxygenase 2; IFN-γ, interferon gamma; IL, interleukin; NF-κB, nuclear factor kappa-B; PGE2, prostaglandin E2; ROS, reactive oxygen species; TNF-α, tumor necrosis factor.
